# Diagnosis of Ovarian Vein Syndrome (OVS) by Computed Tomography (CT) Imaging: A Retrospective Study of 11 Cases

**DOI:** 10.1097/MD.0000000000000053

**Published:** 2014-07-25

**Authors:** Ruizhi Wang, Yan Yan, Songhua Zhan, Litao Song, Weihua Sheng, Xu Song, Xiaolin Wang

**Affiliations:** Shanghai Institute of Medical Imaging (RW, XW); Department of Interventional Radiology (RW, XW), Zhongshan Hospital, Fudan University; Department of Radiology (RW, SZ), Shuguang Hospital Affiliated to Shanghai University of Traditional Chinese Medicine; Department of Obstetrics and Gynecology (YY), Shanghai First Maternity and Infant Hospital; Department of Radiology (LS, WS); Department of Urology (XS), Shanghai Seventh People’s Hospital, Shanghai, China.

## Abstract

This article aims to explore the characteristics of computed tomography (CT) images of ovarian vein syndrome (OVS). The approval of the research ethics committee and the written informed consent of the patients were obtained. The CT images of 11 patients who had been diagnosed with OVS were retrospectively analyzed. All patients were examined with CT urogram, both plain CT scans and enhanced CT scans (including arterial phase, venous phase, and secretory phase). The datum was pulled into a computer workstation for post-processing. Ureteral obstruction at the position and ureteral dilation above it, where the ovarian vein crosses over the ureter, were found in all 11 patients. In addition, 4 patients presented with right upper ureteric calculi, 10 with right renal calculi (including 8 patients with multiple renal calculi that also had obvious uronephrosis), and 2 with a urinary calculus or cystolith. The diameter of the ovarian vein in them ranged from 5 mm to 13 mm. Varicose veins around the uterus were found in 2 patients, and the diameter of the left ovarian vein was larger than 7 mm in 1 patient.

In conclusion, analysis of CT images is a vital method in diagnosing OVS.

## INTRODUCTION

Ovarian vein syndrome (OVS) is a rare condition caused by varicose, dilated ovarian veins inducing chronic ureteral obstruction.^1^ The clinical symptoms of OVS include renal colic, uronephrosis, nephroptosis, hypogastralgia, chronic low back pain,^1–5^ and even impaired renal function in some cases. But OVS is difficult to diagnose. Currently, the most important diagnostic method is imaging examination. To date, limited studies have reported the diagnosis of OVS.^2–7^ In this study, datum of patients diagnosed with OVS that received computed tomography urogram (CTU) in Shanghai Seventh People’s Hospital, Shanghai, China, between August 1, 2008 and July 1, 2012 were analyzed.

## PATIENTS AND METHODS

### Patients

This retrospective study was approved by the research ethics committee of the Shanghai Seventh People’s Hospital. All patients participating gave written informed consent. Eleven female patients with the mean age of 51 years (ranging from 27 y to 76 y) were included in the present study. While all patients had a history of regular menstruation, 8 of them presented with amenorrhea. Out of the 11 patients, 10 were married and had given birth to 1 or more children. Nine of the 11 patients were admitted for renal colic, low back pain, or urinary tract infection (UTI) that was manifested as dysuria, urinary frequency, and urgency. One patient was admitted because of listlessness, weakness, lower eyelid swelling, pretibial edema, and renal insufficiency. Two patients were admitted for uronephrosis as diagnosed by ultrasonic examination in health checks. All 11 patients had a history of UTI (either presenting with symptoms of UTI or had been previously treated for UTI) and symptoms of pelvic congestion syndrome (PCS), which included extensive chronic pelvic pain, dysmenorrhea, and fatigue. Ten out of the 11 patients had a history of renal colic or lower back pain, and 4 had increased levels of serum creatinine and blood urea nitrogen (BUN).

### CT Scanning

GE Lightspeed 16 or GE Lightspeed 64 multilayer spiral computed tomography (CT) (General Electric Company, Fairfield, Connecticut) scanning was performed on all 11 patients. The parameters of the volume CT scanning were as follows: tube voltage was 120 kV; tube current was 200–250 mAs; slice thickness was 5 mm; the pitch was of 1.375 mm/r; 0.8 second was set for the tube to rotate 1 cycle; and the scan matrix was 512 × 512 matrices. The CT scan began at the feet, and the scanning covered the area from the renal upper pole to the pelvic floor. Both plain CT scans and enhanced CT scans (including arterial phase, venous phase, and secretory phase) were performed for all patients. CT number monitoring scanning was used for the arterial phase, followed by the venous phase scanning after an interval of 30 seconds. After another interval of 7–10 minutes, the secretory phase scanning, which covered the area from the lower pole of the 11th thoracic vertebra to the pelvic cavity, was performed. A total of 80–100 mL of Ultravist (300 mgI/mL) (Bayer Schering Pharma AG, Berlin, Germany) was used as the contrast agent, which was injected with the velocity of 2.5–3 mL/second. A GE ADW4.5 workstation (General Electric Company) was used for the post-processing of the images, particularly the multiple planar reconstruction (MPR) and curved plane reconstruction (CPR) of the venous phase images.

## RESULTS

### Clinical Findings

Nine of the 11 patients had obvious symptoms of urology system, which just were renal colic, lower back pain, and UTI’s manifestations as dysuria, urinary frequency, and urgency. Ten had a history of renal colic or low back pain. All patients had a history of UTI (either presenting with the symptoms of UTI or had previously been treated for UTI) and symptoms of PCS (including extensive chronic pelvic pain, dysmenorrhea, and fatigue). Two patients once had received operation of kidney stone and 3 had extracorporeal shock wave lithotripsy. One patient had clinical presentations of renal insufficiency, which were listlessness, weakness, lower eyelid swelling, and pretibial edema. Her creatinine was 148.4 µmol/L (normal standard: 45–90 µmol/L) and BUN was 12.4 mmol/L (normal standard: 2.86–7.14 mmol/L). Other 3 patients had slightly increased levels of creatinine and BUN. Gross hematuria or microscopic hematuria occurred in 9 patients; 2 patients were diagnosed for uronephrosis by ultrasonic examination in health checks without any abnormal symptoms then.

### CT Findings

Right renal calculi were found in 10 out of the 11 patients, including 8 patients with multiple renal calculi, obvious uronephrosis, increased kidney volume and decreased renal parenchyma thickness. Right upper ureteric calculi were found in 4 patients, and urinary calculus or cystolith were found in 2 patients. The varicose right ovarian vein, the mean diameter of which was 9.4 mm (ranging from 5 to 13 mm), crossed over and compressed the ureter in all 11 patients. And the diameter of the left ovarian vein was larger than 7 mm in 1 patient. Varicose veins around the uterus were found in 2 patients.

### Representative Cases

#### Case 1

**FIGURE 1:**
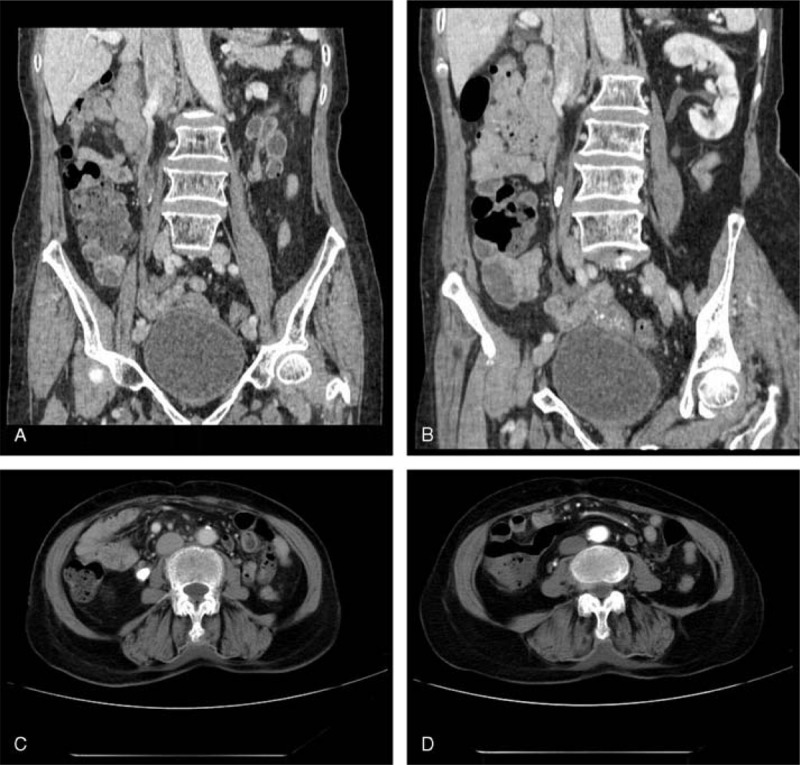
Case 1. (A,B) MPR and (C,D) transverse scanning images display ureterostenosis of the right ureter at the level of the lower margin of the 4th lumbar vertebral body. (B,C) Dense stones and ureterectasia found at and above the place of ureterostenosis, respectively. (C,D) Dilated circuitous ovarian vein nearby that crosses over the ureter is displayed (white arrow indicates the ureter and black arrow indicates the ovarian vein). MPR = multiple planar reconstruction.

A 57-year-old woman who had conceived 4 times and given birth to 3 children was admitted as a result of suffering from listlessness, fatigue, and gross hematuria since 2 days. The patient had a history of right renal calculi and had undergone surgery for this condition. Clinical examinations showed that the patient was listless and answered questions very slowly. The patient’s caretakers reported that the patient had presented with lower right back pain for more than 2 days, a fever, urinary frequency, and dark red urine. Blood examinations showed increased white blood cell counts and neutrophils as well as anemia. Increased creatinine and BUN level, as well as hyponatremia, were also found. Ultrasonic examination revealed significant expansion of the right renal collecting system with multiple renal calculi and right upper ureteral calculi with ureterectasia and hydroureter. CT examination showed the following: severe hydronephrosis of the right kidney; multiple calculi in the renal pelvis; decreased thickness of the renal parenchyma; lower dyeing speed in the right kidney compared with the left kidney; and delayed entering of the contrast agent into the renal pelvis. Ureterostenosis of the right ureter caused by the compression of the enlarged vessels was shown at the level of the lower margin of the 4th lumbar vertebral body. Dense stones and ureterectasia were found above the position of ureterostenosis, respectively. The patient was diagnosed with OVS following surgery (Figure 1).

#### Case 2

**FIGURE 2:**
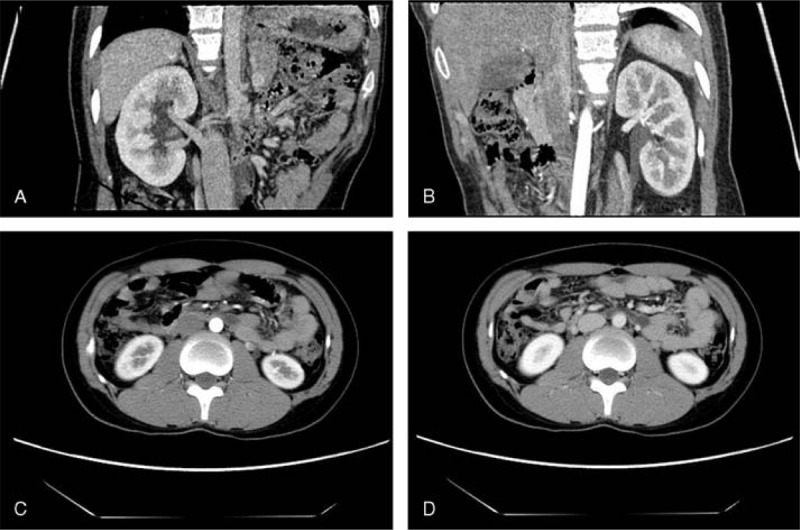
Case 2. (A,B) MPR and (C,D) transverse scanning images display ureterostenosis of the bilateral ureter at the level of the 3rd and 4th lumbar intervertebral space, and ureterectasia above the level of ureterostenosis. A nondilated or circuitous ovarian vein is found crossing over the ureter. Severe uronephrosis is also displayed (white arrow indicates the ureter and black arrow indicates the ovarian vein). MPR = multiple planar reconstruction.

A 27-year-old woman who was unmarried was admitted for bilateral hydronephrosis that was found during a physical examination. Upon admission, the patient was in a healthy condition but, however, suffered from a history of dysmenorrhea. This dysmenorrhea resulted in increased menstrual blood flow and lower abdominal pain and swelling during the menstrual period that was more severe at night or after standing for a long time. CT examination showed mild hydronephrosis in both kidneys, ureterostenosis of the bilateral ureter caused by compression by the enlarged vessels at the level of the 3rd and 4th lumbar intervertebral space, and ureterectasia above the ureterostenosis. The patient was diagnosed with OVS following surgery (Figure 2).

## DISCUSSION

### Ovarian Veins Anatomy

The ovarian veins originate from the ovaries as 5–6 pampiniform plexus that travel behind both sides of uterus body, join the ovarian vein, and then travel along the ovarian artery. The 2 ovarian veins travel upward along the psoas muscles. The right ovarian vein joins the inferior vena cava, while the left ovarian vein joins the left renal vein.^4^

### Pathogenesis

OVS was first reported by Clark^1^ in 1964 whereby the cases of 129 patients with OVS were reported. Few cases have since been reported.^3–6^ According to Clark, OVS is caused by the compression of the ureter by dilated ovarian veins induced by pelvic congestion or an enlarged uterus during pregnancy.^1^ But in 1951, Hodgkinson^8^ reported that while ovarian vein flow can increase 60-fold and that the diameter of the ovarian vein can increase 3-fold, this did not result in compression of the ureter. In addition, a more recent study conducted by Southwell and Bourne^9^ in 1971 showed that when the ovarian vein was fixed and manually dilated, no ureteral obstruction was found.

Together, the earlier report by Hodgkinson^8^ and the later study by Southwell and Bourne^9^ suggest that substantially increased venous pressure causes dilation of the ovarian veins and compresses the ureter thus resulting in OVS. Therefore, these reports explained that although the pregnant women have different levels of ovarian vein dilation, but OVS incidence is not high. This might be because of the threshold pressure that most pregnant women do not reach to cause OVS.

In the current study, the diameters of the right ovarian veins of several patients were 5–13 mm greater than the average diameter. Some right ovarian veins were not significantly dilated, demonstrating that it is the total increase in venous pressure that causes ureteral obstruction. All 11 patients in the current study presented with right OVS (including 1 with both left-side and right-side OVS). As the right ovarian vein joins the inferior vena cava at an acute angle, the venous pressure of the right ovarian vein is generally higher than that of the left.

In addition, alterations of the levels of sex hormones have been regarded as significant factors in the development of OVS. Several studies have indicated that changes in the levels of circulating estrogen and progesterone may alter the flexibility of blood vessels.^10,11^ Dykhuizen and Roberts^5^ proposed that each ovarian vein contains only 1–3 pampiniform plexus, which mostly lose their functions following childbirth, causing dilation and venosclerosis of the ovarian vein. Furthermore, these pampiniform plexus of ovarian veins induce surrounding ureteral fibrovascular embedment and compression of the ureter resulting in OVS. Interestingly, in the present study, a particular patient, referred to as case 2, had not undergone childbirth even though bilateral OVS was found. This again suggests that the occurrence of OVS differs greatly from patient to patient. Consequently, the current retrospective study suggests that a congenital factor may also play a role in the development of OVS.

### Clinical Manifestations

Dilated ovarian veins are capable of compressing the adjacent ureter thus inducing chronic obstruction and causing multiple calculi and recurrent pyelonephritis.^12^ The major clinical symptoms include dysuria, urinary frequency and urgency, constant urinary requirements, gross hematuria, renal colic, lower back pain, lower abdominal and pelvic pain, and dysmenorrhea. The presence of chronic ureteral obstruction, urinary infection, and calculi may occur repeatedly in patients, resulting in patients suffering from the disease for a long period of time, requiring several surgical procedures, or developing renal dysfunction.

### CT Image Features

The existence of chronic ureteral obstruction may result in severe hydronephrosis and hydrocalycosis. In addition, the dilated renal pelvis and renal calyx can compress renal parenchyma resulting in decreased renal parenchyma thickness. The kidney volume, however, is generally substantially increased. High-density shadows (higher than 100 Hu during the CT number) suggest that multiple calculi are displayed in the renal pelvis and calyx. We have found that ureterostenosis caused by compression at the level of the 3rd and 4th lumbar intervertebral space is common in this study. Ureterostenosis commonly presents as a beak-like shape and the ureteral wall appears soft without the bulge and sudden truncation. Calculi in the dilated ureter above the ureterostenosis is easily detected, particularly at the position of ureterostenosis, indicating the need for emergency surgery. Recurrent ureteral infections caused by ureteral obstruction may, in turn, cause thickening of the ureteral walls above the ureterostenosis, as displayed in the enhanced CT images in the current study. The most direct indication of OVS in CT imaging is vessels that cross over the ureter from the outer and above the ureter, to the inner and below the ureter, causing ureterostenosis at the level of the 3rd and 4th lumbar intervertebral space. Enhanced CT scanning may provide conclusive evidence that the above-mentioned vessel is indeed ovarian vein. In general, ovarian vein enlargement is defined as a an ovarian vein diameter ≥7 mm^13^ or >8 mm.^14^

### Diagnosis for OVS and PCS

Previous studies have demonstrated a significant association between OVS and PCS because of a similar pathology^15^ (increased venous pressure causing dilation of ovarian veins). They are all treated with venous embolization or ligation (we had treated all 11 patients with laparoscopy or laparotomy). Many ramus anastomosis exist between ovarian veins and uterine or tubal veins that form a venous circulation. Similarly, these veins also connect with vesical and rectal venous plexus. Consequently, any changes in any of the veins affect the other veins, resulting in OVS being accompanied by PCS. In the present study, all 11 patients presented with the symptoms of PCS (including extensive chronic pelvic pain, dysmenorrhea, and fatigue).

It is, however, vital that differential diagnosis be performed to distinguish OVS and PCS from chronic pelvic inflammatory disease and other diseases that may cause chronic pelvic pain. Comparing with OVS, PCS is not only attributed to dilated ovarian veins but also to a manifestation of dilatation of the entire anastomotic network of pelvic veins (including ovarian, uterine, and iliac), even often with distal extension to involve lower limb veins.^16^ Meanwhile, the pain of PCS is cyclical and associated with menstrual cycle, worse on standing and alleviated on lying down, and dyspareunia and postcoital pain is probable to exist.^17^ Conversely, OVS is mainly characterized by a series of urinary symptoms.

The application of CT scanning plays an essential role in the diagnosis of PCS. Diagnostic criteria for CT scanning in the diagnosis of PCS proposed by Coakley et al^18^ includes: the existence of ipsilateral parauterine veins with at least 4 different diameters, or at least 1 of the veins with a diameter >4 mm; or the diameter of the ovarian vein >8 mm. Coakley et al^18^ also proposed that the purpose of CT scanning is to accurately locate the varicose veins and to identify the possible causes of PCS. Likewise, we are able to make a conclusive diagnosis for OVS by CT and its features are those that are mentioned above.

In some cases of OVS, only mild pyelectasis and hydronephrosis are found. In addition, only some patients with OVS present with mild ureteral dilation above the ureterostenosis. Radiologists unfamiliar with OVS often provide a diagnosis of hydronephrosis and, however, fail to clarify the causes of hydronephrosis, particularly in cases with calculi. Occasionally, radiologists only notice the calculi but overlook the ureterostenosis caused by the compression of the ovarian vein below the calculi. This results in misdiagnosis of the disease, thus affecting treatment options. Ideally, radiologists should review and compare the plain and the enhanced CT images of the arterial, venous, and secretory phases to exclude the “false positive” result caused by ureteral peristalsis. In addition, MPR, particularly CPR of the images, with the center positioned where the ovarian vein crosses the ureter, should be performed to clearly display the anatomical relationship between the vein and the ureter.

## CONCLUSION

In summary, increased knowledge of OVS would substantially improve diagnosis of OVS. Because a disproportionate amount of importance to various urinary symptoms and related CT features is assigned usually, we cannot get a comprehensive diagnosis for overlooking several other symptoms that the changes of the ovarian veins that travel along the ureter. Unfortunately, in many cases that have undergone surgery or other treatments several times, misdiagnosis of OVS has been because of concluding that ureteral obstruction was a result of calculi alone. Therefore, recurrent UTI, calculi, chronic pelvic pain, amenorrhea, and/or dysmenorrhea in women should be considered as serious risks of OVS. CTU can provide comprehensive information for the conclusive diagnosis by only 1 image examination.
